# Music to Her Ears? Male Mice Sing an Ultrasonic Tune

**DOI:** 10.1371/journal.pbio.0030420

**Published:** 2005-11-01

Readers of a certain age may remember Mighty Mouse singing, “Here I come to save the day!” as he raced in to save the innocent from evildoers. Fanciful as his cartoon antics may have seemed at the time (it first aired in the 1950s), the TV show's creators weren't so off the mark. There's still no evidence of superhero behavior in real mice, but in a new study, Timothy E. Holy and Zhongsheng Guo show that mice have a gift for song.

Much of what we know about the biology of song and song learning comes from research on songbirds, but birds are difficult subjects for genetic studies. Song commonly figures in courtship rituals among birds, insects, and frogs, but such behavior in mammals had been restricted to whales, bats, and humans. Evidence of similar behavior in the mouse—a long-established genetic model, often referred to as the pocket human—could open whole new avenues of research into the genetic contributions to song and song learning.

To a lay person, the vocal stylings of rodents appear restricted to squeaks and chatter. But mouse social encounters prompt many vocalizations; some are audible to humans, such as the distress calls of pups, and some aren't, such as the ultrasonic calls of males presented with females or urine pheromones. Previous studies characterized the situations that prompt these vocalizations, but did not detail their acoustics. In this study, Holy and Guo went beyond the conditions that prompt rodent discourse to focus on the sounds themselves. Far from random patter, male ultrasonic calls contain complex passages with long sequences composed of diverse syllable types. [Fig pbio-0030420-g001]

**Figure pbio-0030420-g001:**
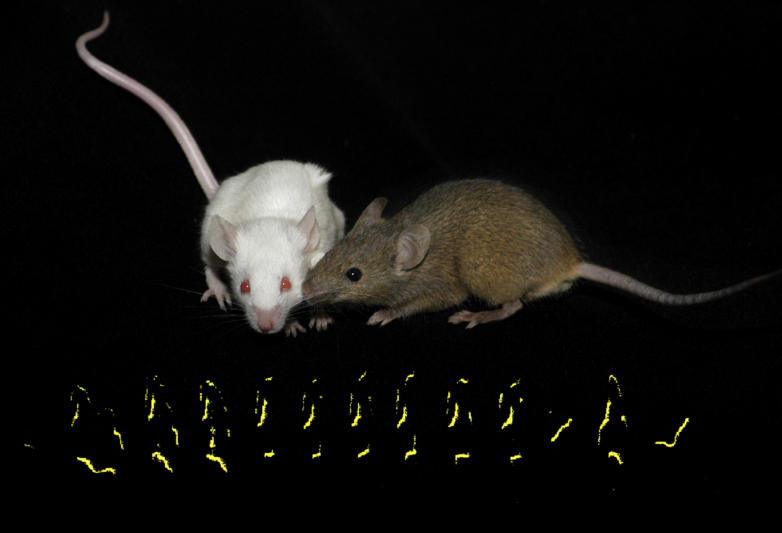
Male mice serenade potential partners with ultrasonic song

The authors used cotton swabs coated with either female mouse urine, male mouse urine, or a combination of the two to elicit the male mouse's ultrasonic sounds, and then recorded their vocal responses. The authors manipulated the recordings to hear the ultrasonics. One approach used a slow playback (at one-sixteenth of the recorded speed), but this distorted the temporal structure of the sounds, and the calls sounded like low, intermittent whistles. The other dropped the pitch to an audible level without interfering with the time sequence—the pitch-shifted recording sounds remarkably like birdsong. (To listen, go to DOI: 10.1371/journal.pbio.0030386.sa004.) To bolster this subjective conclusion, the authors then undertook a quantitative analysis of the sounds.

The males produced rapid “chirp-like” syllables of varying duration, spaced at about ten syllables per second, with a burst of closely spaced syllables followed by periods of silence. Some of the syllables showed sudden, significant changes in frequency (or pitch)—all in keeping with previous reports. To determine whether these frequency jumps, or pitch changes, followed a stereotyped pattern or occurred randomly, the authors first analyzed a set of 750 syllables produced by one mouse in a single 210-second trial. They identified discrete clusters of pitch changes, including two clusters with stereotyped jumps to or from a low frequency, which they called low jumps. Repeating the trial and analysis with 45 different mice produced similar results, indicating that the pitch changes are a universal feature of mouse ultrasonic vocalizations.

These pitch jumps formed three distinct categories, which together with two other techniques—borrowed from previous research in birdsong, speech, and pattern recognition—confirmed that syllables are naturally grouped by their pitch changes. The syllables, in turn, occurred in complex sequences that, in some cases, constituted regularly repeated motifs. Syllables with low jumps were repeated in blocks, and phrases tended to be introduced with syllables containing no pitch jumps at all. Since the mice produced multiple syllable types arranged in regular, repeated time signatures, their vocalizations meet the definition of song. Finally, the authors showed that individual males produced songs distinct from those of other males. “The richness and diversity of mouse song appear to approach that of many songbirds,” Holy and Guo write. And just like songbirds, the mice appear to be singing their own tune. Future studies can begin to unravel the physiological basis and mechanics of ultrasonic mouse song—and perhaps decipher the messages encoded in the notes and melody. —*Liza Gross*

